# Mechanisms of muscle atrophy and hypertrophy: implications in health and disease

**DOI:** 10.1038/s41467-020-20123-1

**Published:** 2021-01-12

**Authors:** Roberta Sartori, Vanina Romanello, Marco Sandri

**Affiliations:** 1 Department of Biomedical Sciences, University of Padova, via Ugo Bassi 58/b, 35121 Padova, Italy; 2 Veneto Institute of Molecular Medicine, via Orus 2, 35129 Padova, Italy; 3 Myology Center, University of Padova, via Ugo Bassi 58/b, 35121 Padova, Italy; 4 Department of Medicine, McGill University, Montreal, Canada

**Keywords:** Cell signalling, Ageing, Energy metabolism, Skeletal muscle

## Abstract

Skeletal muscle is the protein reservoir of our body and an important regulator of glucose and lipid homeostasis. Consequently, the growth or the loss of muscle mass can influence general metabolism, locomotion, eating and respiration. Therefore, it is not surprising that excessive muscle loss is a bad prognostic index of a variety of diseases ranging from cancer, organ failure, infections and unhealthy ageing. Muscle function is influenced by different quality systems that regulate the function of contractile proteins and organelles. These systems are controlled by transcriptional dependent programs that adapt muscle cells to environmental and nutritional clues. Mechanical, oxidative, nutritional and energy stresses, as well as growth factors or cytokines modulate signaling pathways that, ultimately, converge on protein and organelle turnover. Novel insights that control and orchestrate such complex network are continuously emerging and will be summarized in this review. Understanding the mechanisms that control muscle mass will provide therapeutic targets for the treatment of muscle loss in inherited and non-hereditary diseases and for the improvement of the quality of life during ageing.

## Abstract (web summary)

Loss of muscle mass is associated with ageing and with a number of diseases such as cancer. Here, the authors review the signaling pathways that modulate protein synthesis and degradation and gain or loss of muscle mass, and discuss therapeutic implications and future directions for the field.

## Introduction

Being the most abundant tissue (40–50% of the total mass in healthy-weight individual) and the protein reservoir in the human body, skeletal muscles not only control locomotion but they are fundamental for breathing, eating, energy expenditure, as well as for glucose, amino acids, and lipids homeostasis and for maintaining a high quality of life^1^. Consistently, the metabolic adaptations occurring in skeletal muscles are assumed to work as a disease modifier and the quality of muscle mass is an important predictor of mortality^1^.

Skeletal and Cardiac muscle cells are peculiar because the cytoplasm is filled by contractile proteins that are surrounded by organelles, especially the mitochondria and endoplasmic reticulum. Different from all the other cell types, the dense packaging of contractile proteins and organelles does not leave empty space in the cytosol. This organized structure implies that protein and organelle turnover have a major impact on myofiber size and function. In fact, during exercise or anabolic hormonal stimulation, muscles grow because new proteins and organelles accumulate in the cytosol increasing cellular volume, a process named hypertrophy. Conversely, catabolic conditions such as cancer, infections, diabetes, organ failure, or inactivity/disuse promote a net loss of proteins, organelles, and cytoplasm causing shrinkage of the cellular volume, a condition named atrophy. Therefore, the balance between biogenesis/biosynthesis versus removal/destruction defines the size and the function of muscle cells. The pathways that control synthesis versus degradation are regulated by autologous and nonautologous signals, the latter coming from distal organs or from the interstitial cells that surround myofibers, including muscle stem cells.

Before separately considering conditions of muscle growth and muscle atrophy, it is useful to highlight some points.

First, skeletal muscles are heterogeneous with respect to fiber type and metabolic properties and therefore they vary, often drastically, in their response to the same stimulus. For instance, slow muscles, such as the soleus, are less sensitive to starvation compared to fast muscles^2^, while during disuse such as that induced by hind limb suspension, the soleus atrophies faster than glycolytic (fast) muscles^3^. Importantly, the atrophy program diverges within the fast muscles during the same catabolic condition^3^. Therefore, each catabolic condition differs from the others in terms of muscle susceptibility to atrophy as well as induction of an atrophy program and within the same catabolic situation, different muscles activate peculiar atrophy-related programs^3^.

Second, despite the fact that most of the pathways regulating muscle mass, such as Insulin/IGF1 or TGFβ/Activin/BMP, impinge both on protein synthesis and degradation, changes in protein turnover leading to muscle hypertrophy or atrophy do not always proceed according to the simplistic view that muscle growth is consequent to increased protein synthesis and decreased protein degradation, while muscle atrophy results from decreased protein synthesis and increased protein degradation. In fact, exercise does promote the synthesis of new proteins but simultaneously activates autophagy–lysosome and ubiquitin–proteasome-degradative systems^4^. Conversely, during protein breakdown, the amino acids released by lysosome and proteasome directly stimulate mTOR^5^ and therefore, protein synthesis might increase during muscle atrophy.

## Signaling pathways that promote muscle growth

### Insulin/IGF1-AKT-mTOR

**Fig. 1:**
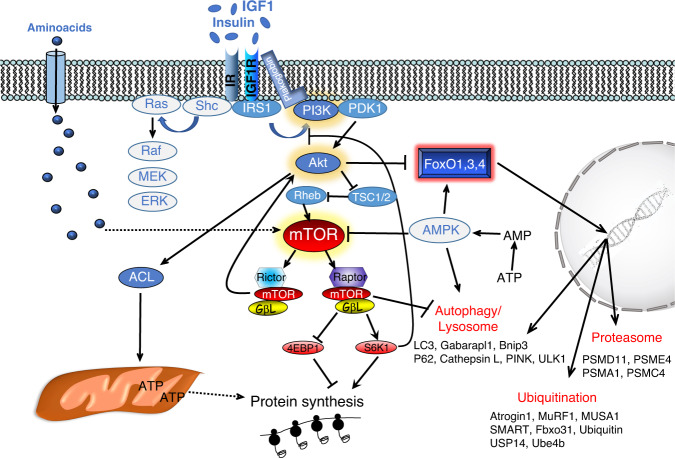
Insulin and IGF1 bind to specific receptors (IR, IGF1R) that activate a cascade of phosphorylation events resulting in enhancement of protein synthesis and inhibition of protein breakdown. Upon receptor activation, IRS1 promotes phosphatidylinositol-3,4,5 triphosphates (PIP3,4,5) generation on the plasma membrane by recruiting the kinase PI3K. Plakoglobin helps the PI3K association to IR. The lipid signal on the plasma membrane promotes AKT recruitment and activation operated by PDK1 and mTORC2 complex. Then AKT positively or negatively modulates a plethora of targets including the mTORC1 complex for protein synthesis, ACL for ATP production, and FoxO for protein degradation. mTORC1 complex is another hub that affects many different biological processes, including autophagy. In fact, mTORC1 phosphorylates and inhibits ULK1. The pathway has several feedbacks that modulate its activity. For instance, activation of mTORC1 blocks IRS1 via S6K1 and consequently, inhibits AKT. AMPK, whose activity is increased by energy stress, is another important modulator of the pathway because blocks mTORC1 and activate FoxO and the autophagy system. Dotted lines depict pathways whose molecular mechanisms and role in adult skeletal muscle have yet to be completely defined.

Insulin and insulin-like growth factor 1 (IGF1) are potent anabolic factors that sustain organism and muscle growth. These hormones bind to specific receptors (insulin receptor and IGF1 receptor) that activate a cascade of phosphorylation events that positively or negatively modulate proteins, enzymes or transcription factors. This pathway regulates protein synthesis, protein degradation, cellular proliferation, and survival as well as glucose uptake and energy production (Fig. 1). While Insulin is produced by the pancreas, IGF1 is synthesized predominately in the liver under the action of growth hormone and acts as a systemic growth factor. However, IGF1 is also produced by extrahepatic tissues where it plays a predominantly autocrine/paracrine role. Muscle-specific overexpression of a locally acting IGF1 isoform in mice showed that localized IGF1 expression sustains muscle growth and regeneration^6^. Among the different IGF1 isoforms, which differ in the N‐terminal signal peptide (Class 1 or 2) and the C‐terminal extension peptide (E‐peptide Ea or Eb), IGF-1Ea is the most powerful in increasing muscle mass and force both in young and aged mice^7^. Insulin and IGF1 are known to activate both the mitogen-activated protein kinase/extracellular signal-regulated kinase (RAS-MAPK-ERK) and the PI3K–AKT-mTOR pathways. However, only a Ras mutant that selectively activates the PI3K–AKT pathway was able to induce hypertrophy of transfected fibers, whereas a Ras mutant acting specifically on the ERK pathway did not^8^. Accordingly, constitutively active AKT results in striking hypertrophy of transfected muscle fibers^9^, with a similar effect being seen using inducible muscle-specific transgenic models^10^.

Insulin/IGF1 signaling impinges on a crucial hub for protein synthesis and degradation that is the kinase mTOR. This kinase integrates multiple stimuli coming not only from hormones but also from cytokines, nutrients, ATP/AMP ratio, and signals to the translation machinery via p70S6K1, which controls the ribosomal protein S6, and factor 4E binding protein 1 (4EBP1), which negatively regulates the ribosomal eukaryotic translation initiator factor 4E^5^. Simultaneously, mTOR inhibits protein breakdown by blocking autophagy via ULK1. The mTOR kinase interacts with several proteins to form two different complexes, the rapamycin-sensitive TORC1 complex, which contains Raptor, and the rapamycin-insensitive TORC2 complex, which contains Rictor (Fig. 1). Genetic approaches identified different functions for these two complexes. While mTORC2 is related to glucose and lipids homeostasis^5^, mTORC1 regulates several anabolic processes, including protein synthesis, ribosome, and mitochondria biogenesis. While muscle-specific Rictor knockout mice do not show any overt phenotype conversely, Raptor- and mTOR-deficient mice display reduced postnatal growth, due to the reduced size of fast but not slow muscle fibers and a progressive muscular dystrophy phenotype^11,12^. Consistently, rapamycin, a specific inhibitor of mTORC1, prevents muscle growth during anabolic conditions^9^. However, recent genetic data suggest that some mTOR actions may be independent of the mTORC1 complex. For instance, inducible muscle-specific deletion of Raptor does block muscle hypertrophy but does not blunt the increase of protein synthesis, revealed by puromycin incorporation, in a mechanical overload model^13^. Conditional deletion of mTOR with a concomitant expression of catalytically inactive mTOR, resulted in a growth rate defect that started after 1 week of age. Importantly, these mice show a more severe muscle loss when compared with the conditional RAPTOR knockout^11^. Despite the profound inhibition of the mTORC1 complex in transgenic mice, the muscles still grow, although to a lesser extent than control. However, both conditional mTOR knockout and mTOR catalytical inactive transgenic mice show myofiber degeneration and myopathic phenotype, suggesting an important pro-survival role of mTOR. Surprisingly, autophagy is not hyperactivated in these mice, as might be expected to occur following inhibition of the mTORC1 complex (Fig. 1), but instead is impaired and this inhibition greatly contributes to the pathological features of these animals^12,14^. A slow and progressive myopathic phenotype was also described in mice that displayed a chronic mTORC1 activation by TSC1 inhibition^15^. Consistent with mTOR hyperactivation, the autophagy system was blocked and this impairment contributed to the phenotype of TSC1 knockout^15^. Altogether these findings suggest that mTORC1 has a major role in muscle homeostasis but may not be the exclusive regulator of protein synthesis and that autophagy in muscles is controlled by mTORC1 independent and dependent pathways.

### TGFβ/myostatin/activin/BMP

**Fig. 2:**
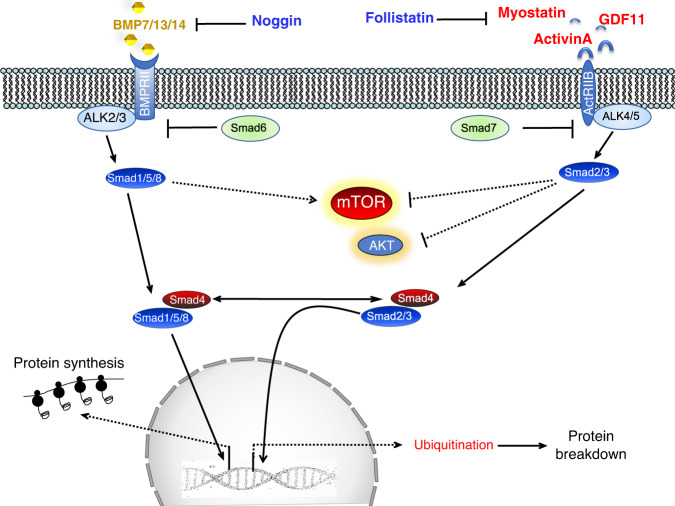
TGFβ superfamily activates two different pools of transcription factors with the opposite function. Myostatin (GDF8), activinA/B, TGFβ, and GDF11 bind to type II receptors like ActRIIB/IIA that activate type I receptors (ALK4/5/7), which phosphorylate and induce Smad2/3 to form a complex with Smad4 and translocate into the nucleus. BMP ligands bind BMP type II receptors like BMPRII but also ActRIIB/A and recruit type 1 receptors such as ALK2/3/6 to phosphorylate and activate Smad 1/5/8 that form a complex with Smad4. The BMP and TGFβ/myostatin/activins ligands are modulated extracellularly by the inhibitory action of cytokines, such as noggin and follistatin. Another level of regulation happens in the cytosol where Smad6 and 7 negatively modulate the BMP and myostatin/activin pathway. Finally, both Smad2/3 and Smad1/5/8 transcription factors regulate mTOR activity, but the underlying mechanism are unclear. Dotted lines depict pathways whose molecular mechanisms and role in adult skeletal muscles have yet to be completely defined.

The second major signaling pathway that controls skeletal muscle growth involves myostatin, a member of the transforming growth factor β (TGFβ) superfamily. The TGFβ superfamily comprises more than 30 secreted ligands with differing selectivity for specific receptor subtypes. Myostatin is the most well-known member of this superfamily, in the muscle field, because of the profound hypermuscularity of Myostatin knockout mice^16^. The Activin/Myostatin/TGFβ group binds plasma membrane-associated activin type IIB and type IIA receptors (ActRIIB/IIA) and TGFβ receptors (TGFβRII) and, through the recruitment and activation of activin receptor-like kinase (ALK)-4, -7, and -5, induce phosphorylation of Smad2/3 to promote the formation of a heterotrimeric complex with Smad4 (Fig. 2). Importantly, just inhibition of Smad2/3 is sufficient to promote muscle growth^17,18^, suggesting that genes involved in protein turnover are the target of these transcription factors. Several findings indicate the presence of cross-talk between myostatin and the AKT/mTOR axis. Indeed, rapamycin treatment or mTOR knockdown revert the hypertrophic effect of myostatin blockade^17,18^.

Another pathway that converges on Smad4 and controls muscle mass is BMP signaling^19,20^. BMP/GDF members preferentially bind to a combination of type II receptors that include BMP type II receptor (BMPRII), ActRIIA, and ActRIIB, before promoting recruitment of type I receptors, such as BMPRIA (ALK3), BMPRIB (ALK6), and ACVR1 (ALK2). These ligand/TypeII/TypeI receptor complexes promote phosphorylation and heterotrimerisation of Smad1/5/8 with Smad4 to affect transcriptional regulation (Fig. 2). Thus, in addition to Smad4, ligands from both superfamily subgroups also conceivably compete for access to some type II receptors. Modulation of the pathway also occurs downstream of the receptors. For instance, the inhibitor proteins Smad6 and 7 prevent receptor-mediated activation of Smad1/5/8 and Smad2/3^21^. Interestingly, specific ablation of Smad4 in the skeletal muscle of mice did not promote hypertrophy but caused atrophy and weakness^19^. The finding that overexpression of the BMP antagonist noggin reverted the hypertrophic phenotype of myostatin knockout mice, strongly argues in favor of genetic epistasis between the activin/myostatin and BMP pathways in muscle. Consistently, follistatin-mediated hypertrophy not only blocks myostatin signaling but simultaneously stimulates Smad1/5/8 activation^19,22,23^. Therefore, the inhibition of myostatin/activins and the consequently reduced levels of phosphorylated Smad2/3 would release Smad4 to interact with phosphorylated Smad1/5/8, to sustain growth and/or counteract atrophy.

### β-adrenergic signaling

Adrenergic signaling is another pathway that regulates muscle mass via its connection with the AKT-mTOR axis. Muscle hypertrophy induced by β2-adrenergic agonists, such as clenbuterol or formoterol, is accompanied by a significant increase in AKT phosphorylation and is completely blocked by rapamycin^24^. Recent data suggest that β2-adrenergic signaling, at least partially, impinges on insulin/IGF1 receptor signaling and not on the ERK1 pathway^25^. Indeed, genetic and pharmacological inhibition of insulin receptor, IGF1 receptor, PI3K, and AKT abolished the anti-proteolytic effects of the β-adrenergic agonist, formoterol, while the ERK1/2 inhibitor, U0126 did not.

### Novel and emerging pathways that need consolidation: FGF/desmosomes/Zinc ions

It has been recently reported that administration of FGF19 promoted muscle hypertrophy and increased grip strength without AKT activation but with ERK stimulation^26^. This finding is in contrast with another report showing that FGF21 is sufficient and required to induce muscle loss^27^. FGF19 (FGF15 in the mouse) FGF21, and FGF23 are unique among FGF ligands because they do not bind heparan sulfates but interact with α- or β-klotho proteins that work as co-receptors or co-ligands of FGFR. Since FGF19 and FGF21 bind to β-klotho and activate the same receptors (FGFR1-4), it is expected that they should elicit the same function.

A modulator of insulin receptor activity was recently identified in plakoglobin, a desmosomal component that binds the insulin receptor and PI3K subunit p85. Overexpression of plakoglobin enhances PI3K–AKT-FoxO signaling and promotes muscle growth^28^. Another interesting connection is between zinc ions and muscle growth. Metallothioneins are zinc-binding proteins that belong to atrogenes (see below). Inhibition of these proteins releases zinc ions which trigger hypertrophy. Mechanistically, knocking down as well as genetic ablation of metallothionein 2 promote muscle growth in mice probably via AKT-mTOR axis^29^. Conversely, the increase of the metal-ion transporter ZRT- and IRT-like protein 14 (ZIP14), which controls the uptake of Zn ions, has been shown to promote muscle loss. ZIP14 is upregulated by TNF-α and TGF-β cytokines and ZIP14-dependent zinc accumulation induces myosin heavy chain loss, while muscle-specific deletion of ZIP14 prevents muscle loss in tumor-bearing mice^30^.

## Metabolic regulators that sustain muscle growth

Because protein synthesis consumes almost 30% of the total ATP pool, the mitochondria/energy production system is required to sustain anabolism. Consistent with the hypothesis that metabolic changes are permissive for muscle growth, the mitochondrial calcium uniporter (MCU), the highly selective channel responsible for mitochondrial Ca2+ uptake, has been found to control glucose oxidation and to positively regulate myofiber size in physiological conditions. Importantly, overexpression of MCU counteracted pathological loss of muscle mass, probably via PGC1α4 upregulation^31^. PGC1α4 is a spicing variant of PGC1α, the master gene that controls mitochondrial function. However, despite the fact that PGC1α transgenic mice do not show muscle growth and do not display an enhancement of protein synthesis, the splicing isoform 4 (PGC1α4) was found to promote muscle hypertrophy^32^. Mechanistically, PGC1α4 splicing variant promotes the expression of IGF1 via the upregulation of G protein-coupled receptor (GPR) 56^33^.

Besides mitochondria, fuel utilization is also a determinant for muscle growth. For instance, the anabolic action of IGF1 requires the activity of ATP citrate lyase (ACL), a cytosolic enzyme that catalyzes the conversion of mitochondrial-derived citrate into oxaloacetate and acetyl-CoA. Indeed, ACL is phosphorylated and activated by AKT resulting in an improvement of mitochondrial respiration and ATP production^34^.

Amino acids, especially the branched ones, are well-known growth-promoting agents because they directly stimulate mTOR^5^. Importantly, metabolites of amino acids, which are also intermediates of mitochondria TCA cycle (e.g., α-ketoglutarate) and are sensed by specific receptors like GPR91, also impinge on AKT-mTOR axis^35^.

### Novel and emerging pathways that need consolidation: microbiota/UBR4/perilipins

The gut microbiota generates several metabolites including amino acids like glycine and alanine and the neurotransmitter precursor choline that can positively regulate muscle mass. Indeed, germ-free mice show an increased plasma level of glucocorticoids, decreased IGF1 and PGC1α expression, alteration of mitochondrial function, and induction of catabolic enzymes for branched-chain amino acids (BCAAs) leading to poor quality of muscles^36^.

Ubiquitin ligases have been associated with atrophy. However, a recent RNAi screening for the ubiquitin ligases that control muscle mass identified UBR4 as a critical one for hypertrophy^37^. UBR4 induces hypertrophy via ubiquitination and degradation of a core set of target proteins, including the HAT1/RBBP4/RBBP7 histone-binding complex, that control histone acetylation and expression of growth-promoting genes.

Finally, the type of fuel for ATP synthesis in mitochondria may be also an important step for muscle growth. For instance, alteration in lipid composition due to the downregulation of the lipid-associated protein Perilipin2 is sufficient to induce hypertrophy and prevent muscle atrophy after denervation^38^. Interestingly, this type of hypertrophy seems to be largely mTOR-independent because rapamycin treatment did not prevent muscle growth after Perilipin2 knockdown. The inhibition of Perilipin2 caused a reduction of triglyceride and ceramides, which are established negative regulators of muscle trophism.

## Muscle atrophy

The comparison of the gene expression profiles of muscles from different catabolic conditions led to the identification of a common set of genes that were named atrogenes. Since all these diseases have in common muscle atrophy, these genes are thought to be important for protein loss. Among these atrophy-related genes there are several that belong to the major cellular degradation systems, the ubiquitin–proteasome and autophagy–lysosome. The transcriptional-dependent induction of these atrogenes might be required to replenish the enzymes/proteins that are lost during the enhanced protein breakdown. The ubiquitin ligases, the rate-limiting enzymes during the ubiquitination process, undergo autoubiquitination^39^, and therefore, an increased ligase activity during catabolic conditions would inevitably amplify its autoubiquitination and its proteasomal-dependent degradation. Therefore, the transcriptional upregulation is particularly important mostly to replenish the loss of the ubiquitin ligase proteins. Similarly, several autophagy-related proteins, like LC3, Gabarap, p62, NBR1, Optineurin, PINK1, Parkin, BNIP3, and BNIP3l/Nix, are entrapped into the autophagosome when the vesicle is formed and therefore are destroyed upon a fusion of the autophagosome with the lysosome^40,41^. Thus, it would appear that increased expression might be required to restore their levels and sustain autophagy flux in catabolic conditions. This concept should be considered when protein expression does not match with the transcript level.

### Ubiquitin–proteasome system

The first ubiquitin ligases that were identified to play a role in muscle loss were Atrogin-1/MAFbx and MuRF1. These two E3s are strongly upregulated in all the catabolic conditions so far tested in animals and are considered the master genes of muscle atrophy^39^. MuRF1 ubiquitinates several muscle structural proteins, including troponin I, myosin heavy chains, actin, myosin-binding protein C, and myosin light chains 1 and 2^42^. In comparison, Atrogin-1 substrates seem to be involved in growth-related processes or survival pathways^42^. However, data from knockout mice do not sustain the exclusive role of these E3s in muscle loss. MuRF1 knockout  mice are not protected from denervation-induced muscle loss at 7 days and only partially protected (around 30%) at 14 days^39^. Moreover, the absence of MuRF1 does not protect from fasting or microgravity induced atrophy, while muscle mass is spared following glucocorticoid treatment^43,44^. Finally, MuRF1 inhibition prevents age-related muscle loss but not the decrease in muscle force^45,46^. Inhibition of Atrogin-1 does not block denervation- and glucocorticoids-induced muscle loss^19,44^ and does not protect from aging sarcopenia^46^.

Altogether these observations open the field for the identification of other ubiquitin ligases that contribute to the degradation of sarcomeric proteins. Trim32 has been found to control the thin filaments (actin, tropomyosin, troponins), the Z-band protein α-actinin, and desmin^47^. Moreover, TRIM32 has been recently linked to autophagy because it enhances ULK1 activity via the recruitment of an unanchored Lys63-linked polyubiquitin chain, an unusual way to regulate protein function (Box 1)^48^.

Another E3 ubiquitin ligase that was found to play a critical role in ULK1 regulation and in muscle wasting is TRAF6^49^. This E3 ligase mediates the conjugation of Lys63-linked polyubiquitin chains to target proteins. Lys48-linked polyubiquitin chains are a signal for proteasome-dependent degradation, while Lys63-linked polyubiquitin chains play other roles, such as regulating autophagy-dependent cargo recognition by interacting with the scaffold protein p62 (also known as SQSTM1)^40^. Notably, muscle-specific TRAF6 knockout mice are resistant to muscle loss induced by cancer, denervation, and starvation^50^.

Box 1 ubiquitination process at a glance In the ubiquitin–proteasome system, proteins are targeted for degradation by the 26S proteasome through covalent attachment of a chain of ubiquitin molecules. Different classes of enzymes, named E1, E2, and E3, are involved in protein ubiquitination. The E1 is the ubiquitin-activating enzyme that hydrolyses ATP to bind ubiquitin and is encoded by only one gene. E2 receives ubiquitin from the E1 and brings it to the E3 complex. The E3 enzymes recognize the target, assemble the complex with the E2 enzyme, and catalyze the transfer of the ubiquitin from E2 to the substrate. This reaction is the rate-limiting step of the ubiquitination process, which affects the subsequent proteasome-dependent degradation. Once the protein is ubiquitinated it is docked to the proteasome for degradation, unless the polyubiquitin chain is removed by the de-ubiquitinating enzymes. Different E2–E3 pairs function in the degradation of different proteins, and the specificity of the E3s for specific groups of proteins provides exquisite selectivity to this degradation process. The human genome contains around one hundred E2 genes and almost one thousand E3 genes. This great level of heterogeneity is involved in the precise regulation of different cellular processes. The content of different E2s and E3s varies between tissues and with different physiological conditions, but it is still unknown which specific E2s and E3s normally work in muscle.

### Autophagy–lysosome system

**Fig. 3:**
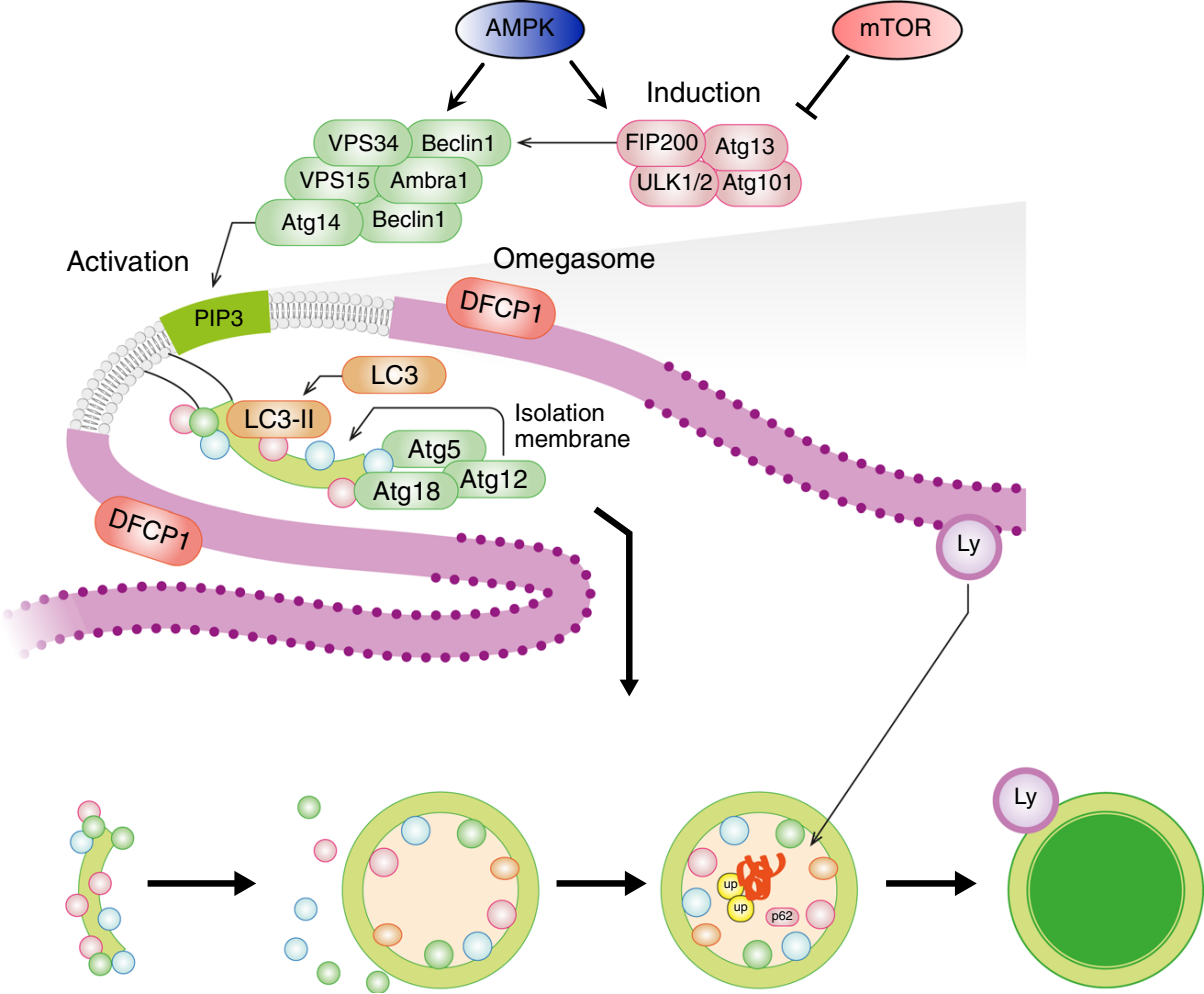
Autophagosome formation is highly regulated and involves many proteins. Assembly of the ULK1-FIP200-Atg13-Atg101 complex primes the induction of Beclin1-VPS34-VPS15 complex to generate phosphatidylinositol-3-phosphate (PIP3) lipids in membranes of different organelles (e.g., endoplasmic reticulum). The ULK1 and the Beclin1 complexes are negatively or positively modulated by mTOR and AMPK, respectively. The PIP3 signal recruits the conjugation systems (LC3/GABARAP and Atg5/Atg12/Atg16) of autophagy that induces the covalent linking of phospholipids with LC3-GABARAP proteins for membrane’s commitment to becoming autophagosomes. Then the committed membrane (phagophore assembly site or isolation membrane) elongates and closes to form a double-layered vesicle (autophagosome) that is docked to the lysosome (Ly) for cargo destruction. The endoplasmic reticulum site where autophagosomes are formed, which is revealed by DFCP1 protein, is named omegasome.

Macroautophagy, hereafter referred to as autophagy, is the other proteolytic system that is activated in catabolic conditions and that is under transcriptional-dependent control. Indeed, some of the autophagy-related genes (Atg) especially the ones related to membrane commitment and cargo recognition belong to the list of the atrogenes. Although autophagy was initially considered a non-selective degradation pathway, the presence of selective forms of autophagy is established (Box 2). Indeed, autophagy can trigger the selective removal of either specific organelles such as mitochondria or proteins, including protein aggregates (Fig. 3). The specificity is elicited by some adaptor proteins such as p62, Bnip3 that simultaneously bind autophagosomes via LC3 and the dysfunctional organelles/damaged proteins/protein aggregates that usually, but not exclusively, are labeled by a polyubiquitin chain. Hyperactivation of autophagy has been found to contribute to muscle loss in a plethora of conditions, including cancer cachexia, fasting, sepsis, critical illness, cirrhosis, chemotherapy, disuse, denervation, COPD^41^. Because autophagy is constantly active in the cell to generate metabolites that sustain cellular core metabolism, to remove damaged components, and to promote repair and stress resistance, an autophagy impairment induces muscle degeneration and weakness^51^. This phenotype is common among different muscle-specific knockout mice for autophagy-related proteins, such as Atg5, VPS15, ULK2, AMPK, and mTOR^15,52–55^. Importantly, specific deletion of Atg7 or overexpression of TSC2 recapitulates the pathological features of aging sarcopenia, such as increased oxidative stress, accumulation of dysfunctional mitochondria, myofiber denervation, atrophy, weakness, and premature death^15,51^. Reactivation of autophagy in aged mice both by expressing Atg7 in skeletal or by treating mice with Urolithin A, a compound that promotes mitophagy, ameliorate muscle mass^51,56^. Importantly, a link between autophagy failure, oxidation of contractile proteins, and impaired force generation has been shown^51^.

Box 2 The autophagy system at a glance The process of autophagy proceeds through several mechanistically distinct steps that include (Fig. 3): (i) regulatory and activating complexes to induce the autophagosome formation, (ii) double-membrane commitment to generate the phagophore assembly site (PAS) or isolation membrane, (iii) membrane elongation to form a complete double-layered vesicle, the autophagosome, that sequesters cellular components, known as the cargo, (iv) autophagosome docking and fusion with the lysosome, (v) degradation of the cargo and release of amino acids, lipids and glucose, (vi) lysosomal rejuvenation and biogenesis.

In the cell, the ULK1-2/Fip200/Atg13/Atg101 complex is an upstream activator of the autophagy system (Fig. 3) and is modulated by nutrients and energy-sensitive pathways, such as mTOR and AMPK. The ULK1 complex is blocked by mTORC1 and is induced by AMPK. This complex promotes the recruitment on the membranes of the Beclin1 complex that contains the class III phosphoinositol-3-kinase Vps34 to produce phosphatidylinositol-3-phosphate on the membrane at the phagophore assembly site (PAS). This activity is coordinated by the interaction of Beclin1 with several other proteins, including Bcl-2, Vps150, UVRAG, Ambra1, Bif-1, Atg14L/Barkor, and Rubicon, to form different protein complexes mediating distinct functions. These two steps are critical for the assembly of the conjugation system on the membranes. The conjugation system works similarly to the ubiquitination system and small ubiquitin-like molecules (LC3, GABARAPL1, GABARABL2, Atg12) are activated and transferred from the conjugation proteins to membranes for their growth and commitment to become a double-membrane autophagosome vesicle.

Thus, these reactions require the recruitment and assembly of different components of the autophagy machinery on phospholipids, but only the ubiquitin-like components LC3, GABARAP are covalently bound to the phosphatidylethanolamine, a phospholipid present both on the outer and inner membranes of the autophagosome. Finally, the autophagosome with the cargo is delivered to the lysosome, and membrane fusion allows cargo degradation by the lysosomal acidic hydrolases and the recycling of the molecules. The lysosomes are then regenerated by the transcription factor TFEB, which is under mTOR regulation and is activated when autophagy is induced^5^.

### Novel and emerging pathways that need consolidation: ubiquitin–proteasome system

MUSA1^19^, previously named Fbxo30, SMART, previously named Fbxo21, and Fbxo31^57^ are novel ubiquitin ligases belonging to the SCF complex family that is induced in atrophying muscles. Among these, inhibition of MUSA1 and SMART in denervated muscles via RNA-interference has been found to reduce the severity of neurogenic atrophy^19,57^.

The E3 ligases so far described are activated during atrophy, but none of these cause atrophy when overexpressed in normal muscles^19^. An exception is the muscle-specific ubiquitin ligase, SOCS box protein 2 (Asb2b)^22^, which was identified to be repressed by Follistatin and to be critical for hypertrophy. In fact, while its inhibition is permissive for growth, its overexpression causes muscle atrophy^22^.

Among the atrogenes, several belong to the proteasome subunits suggesting that proteasome activity could be enhanced by changes in the regulatory subunits. The zinc finger protein ZNF216 binds the 26S proteasome and enhances the proteolytic activity by increasing ATP hydrolysis^58^. Consistently, the absence of nutrients induces PKA-mediated phosphorylation of the proteasome subunits Rpn6, enhancing proteasomal degradation^59^.

Finally, another player is the p97/valosin-containing protein (VCP) ATPase complex whose function is to extract and degrade ubiquitinated proteins from larger structures, including myofibrils^60^. p97/VCP is induced during denervation, and overexpression of a dominant-negative p97/VCP reduces overall proteolysis by the proteasome and lysosome pathways. The activity of p97 is modulated by the methyltransferase-like 21e (Mettl21e) and Meetl21c^61,62^. While Mettl21e negatively regulates 26S proteasome activity, Mettl21c seems to be crucial for autophagy regulation. Interestingly, the two methyltransferases showed different fiber-type specificity being type 2b fibers more affected by Mettl21e ablation^61^ and type 1 by deletion of Mettl21c, which is also preferentially expressed in slow fibers^62^. However, the substrates of methyltransferases are multiple and, consequently, the phenotype of these mice may be p97-independent.

## Signaling pathways that control muscle loss

### FoxOs-atrogenes

After the discovery of the atrogenes, the concept that transcription factors drive muscle atrophy is well-established. FoxO1,3,4 are transcription factors downstream the IGF1/insulin-Akt pathway (Fig. 1) and their inhibition completely spares muscle loss and force drop in fasting, hind limb suspension, immobilization, diabetes, and glucocorticoids treatment^3,57,63,64^. Fine-tuning of FoxOs activity is essential to avoid excessive or defective protein breakdown. Indeed, they are regulated by many different post-translational modifications including phosphorylation, acetylation/deacetylation, ubiquitination, arginine, and lysine methylation. These regulatory changes can either activate or repress FoxO action in response to a variety of stimuli, like oxidative stress and variations in nutrient status. Consequently, many different kinases (e.g., AKT, SGK1, AMPK, p38 MAPK, ERK, JNK, MST1) and acetyltransferase or deacetylase positively or negatively modulate FoxO activity. For instance, p300/CBP-mediated acetylation inhibits FoxO3-dependent atrophy program, while HDAC-mediated deacetylation activates FoxOs^65,66^. Besides post-translational modifications, FoxO recruitment on target genes is also modulated by direct or indirect action with cofactors or with other transcription factors. For instance, JunB, PGC1α, and PGC1β all inhibit FoxO activity in skeletal muscles^2,67,68^, while the glucocorticoid receptor and β−catenin synergize with FoxOs to promote muscle wasting^69^. Finally, an additional level of regulation is the transcriptional upregulation of FoxO gene. In fact, mRNA levels of FoxO members are induced in many catabolic conditions, a process that is transcriptionally regulated by glucocorticoid receptor^70^.

### TNFα-IKK-IkB-NF-kB

**Fig. 4:**
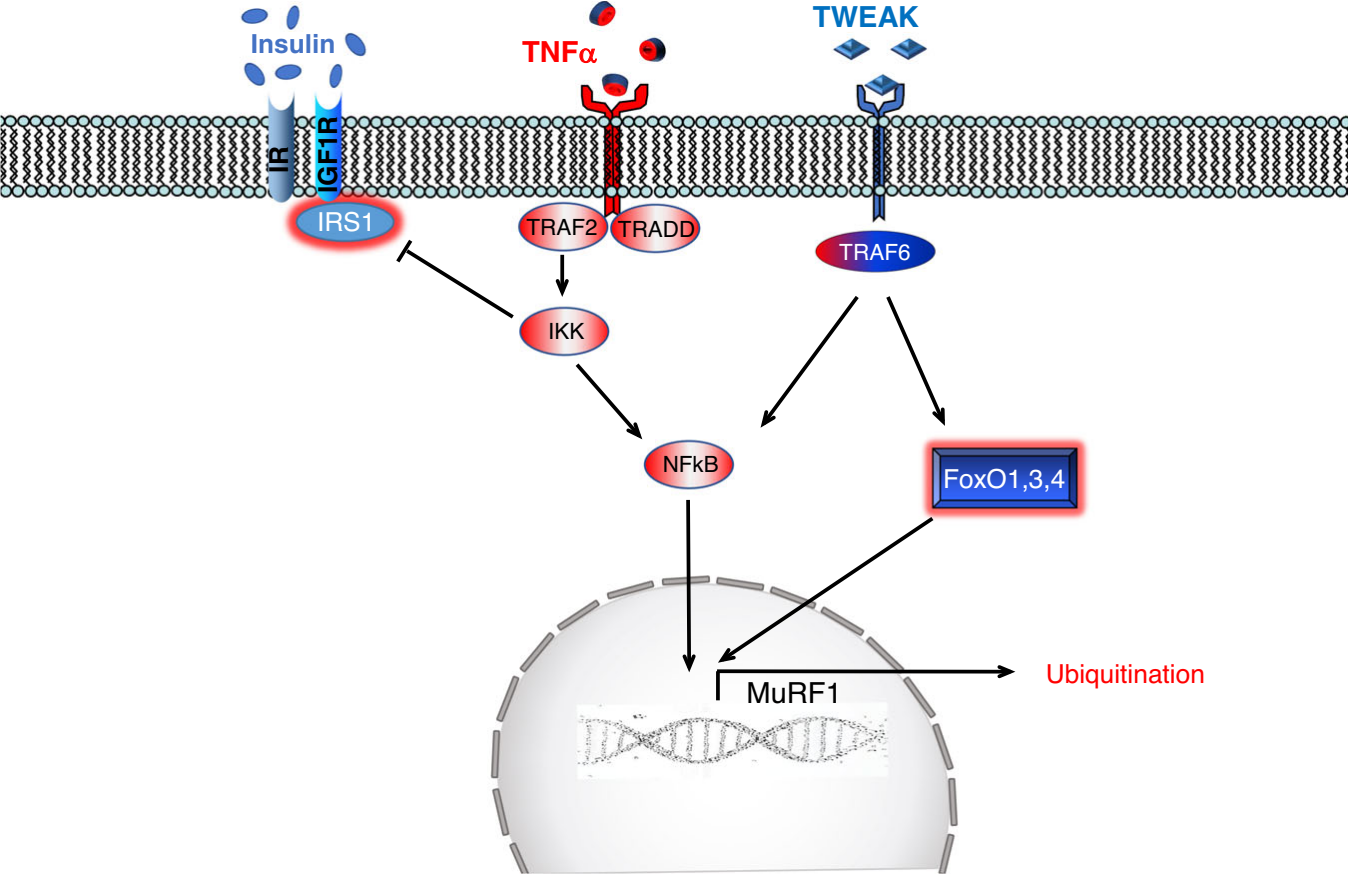
Soluble TNFα binds, induces trimerization, and activates TNF-receptor (TNFR) which recruits several adaptor proteins (e.g., TRAF2) that trigger IKK complex activation. IKK phosphorylates the inhibitory of NF-kB, named IkB, that undergoes proteasomal-dependent degradation leading to NF-kB release and nuclear translocation. IKK activation leads also to inhibition of the insulin pathway by blocking IRS1. Tweak, another member of TNF family, binds to the receptor, named Fn14, which upon activation recruits proteins such as TRAF6 to induce NF-kB as well as FoxO activation.

The NF-κB transcription factor, which is a master regulator of immunity and inflammation, mediates the effect of inflammatory cytokines, in particular, TNF-α and IL6, on muscle wasting. In the inactive state, NF-κB is sequestered in the cytoplasm by the inhibitory proteins IκB (Fig. 4). In response to TNF-α, the IκB kinase complex (IKK) phosphorylates IκB resulting in its ubiquitination and proteasomal degradation, leading to the nuclear translocation of NF-κB and expression of its target genes (Fig. 4). Muscle-specific overexpression of IKKβ in transgenic mice leads to severe muscle wasting mediated, at least in part, by the ubiquitin ligase MuRF1, but not by atrogin-1/MAFbx^71^. NF-kB and the atrophy program are also positively modulated by tumor necrosis factor-like weak inducer of apoptosis (TWEAK), which is a member of the TNF superfamily^72^. TWEAK acts on responsive cells via binding to fibroblast growth factor-inducible 14 (Fn14), a small cell surface receptor. Fn14 is upregulated in denervated muscle allowing NF-κB activation and consequently MuRF1 but not atrogin-1/MAFbx expression^72^. TWEAK knockout mice display less atrophy during denervation as well as reduced NF-κB activation and MuRF1 expression. However, Fn14 does not increase in all the conditions of muscle atrophy, for instance, it is not induced by dexamethasone treatment^72^.

### IL6-JAK-Stat3

**Fig. 5:**
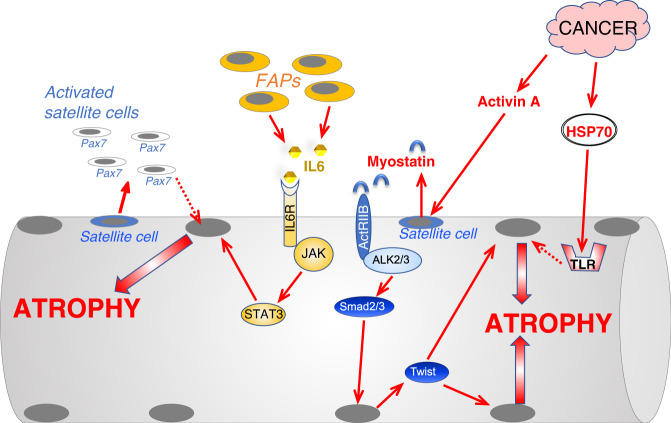
Scheme of the principal pathways induced by the interstitial cells or by cancer that control protein degradation and muscle atrophy. Cancer growth induces hyperproliferation of Pax7-positive muscle stem cells, named satellite cells (SC), that do not fuse with myofiber and triggers an atrophy program whose insights are still unclear. When exposed to an increased TGFβ signaling, satellite cells express Twist that induces myostatin expression and secretion. The release of myostatin causes a second wave in myofibers where Twist and Smad3 cooperate to promote muscle atrophy. Denervation, cause expansion of the fibro-adipogenic precursor (FAP) cells which induce an inflammatory response via IL6 leading to Stat3 activation in muscle and induction of an atrophy program. Cancer derived exosomes deliver HSP70/90 to myofibers that activate TLR4 and an atrophy program. Dotted lines depict pathways whose molecular mechanisms and role in adult skeletal muscle have yet to be completely defined.

The transcription factor Stat3 is activated by the pro-inflammatory cytokines TNFα, IL6, and IL1. IL6 by binding the complex IL6R-gp130 induces the kinase JAK that phosphorylates and activates STAT3 (Fig. 5). Interestingly, cancer and sepsis induce Stat3 phosphorylation in muscles and Stat3 inhibition spares muscle mass in tumor-bearing mice^73^ (Fig. 5). Moreover, overexpression of Stat3 is sufficient to induce muscle atrophy and to upregulate atrogin-1^73^.

### ATF4 and ER stress

Unfolded protein response (UPR) and endoplasmic reticulum (ER)-stress-related pathways have been found to regulate muscle atrophy. The UPR-downstream transcription factor ATF4 promotes muscle atrophy by modulating a subset of atrogenes like Gadd45α and P21. Gadd45α induces the remodeling of chromatin to repress genes involved in anabolic signaling and energy production and to activate pro-atrophy genes including autophagy and caspase-related genes^74^. ATF4 is activated when the chaperone GPR78/Bip is engaged to refold proteins in ER, leading to the activation of the kinase PERK that phosphorylates the initiation factor eIF2α and, ultimately, reduces ribosome assembly and protein synthesis. The decrease of translation allows the synthesis of a subset of mRNA that has multiple open-reading frames, including ATF4. However, loss of function of PERK did not reduce but instead caused muscle loss. Both inducible inhibition or knockdown of PERK in muscle and myotubes resulted in atrophy due to upregulation of autophagy and ubiquitin–proteasome genes^75^. This contradiction may be explained because PERK downstream targets are multiple (e.g., eIF2α, NRF2). More recently, another branch of the UPR, the IRE1-XBP1 pathway, has been linked to muscle mass regulation. IRE1 is also activated by the reduction of GRP78/Bip in ER and induces XBP1 transcription factor. Target ablation in the muscle of XBP1 ameliorates muscle loss in tumor-bearing mice while its overexpression promotes myotube atrophy in vitro^76^.

### Mitochondrial dysfunction

In many catabolic conditions, mitochondrial quality control and the mitochondrial fusion and fission proteins are dysregulated^77^. Consistently, knockout mice for the fusion protein OPA1 or the fission component DRP1 display muscle wasting^77,78^. However, the shape of the mitochondrial network, as well as muscle and animal phenotypes, are different depending on whether the fusion or fission machinery is blocked. Acute inhibition of OPA1 induces oxidative stress, systemic inflammatory response, muscle atrophy, premature tissue senescence, that altogether cause animal death^77^. Conversely, DRP1 ablation alters calcium homeostasis and mitochondria–ER tethering resulting in myofiber degeneration but with normal animal survival^78^. Since mitochondrial respiration is altered in both mice models, a new concept emerges from these findings: that mitochondrial network shape, more than mitochondrial function, is important for muscle mass regulation.

### Novel and emerging pathways that need consolidation: FoxO regulation

Among the different post-translational modifications of FoxOs, it has been recently shown that arginine 188 and 249 methylation by the arginine methyltransferase PRMT6 enhances the catabolic action of FoxO3^79^. An increase of FoxO activity has been reported by the interaction among FoxO and β-catenin, which is regulated by the secreted glycoprotein Dickkopf (DKK3). DKK3 is upregulated in aging and its overexpression caused muscle atrophy, while its downregulation reduced age-related muscle loss but not cancer- or fasting-mediated muscle atrophy. The age-related muscle loss is consequent to β-catenin recruitment on FoxO3-binding sites to enhance the expression of atrogin-1 and MuRF1 expression^69^.

Chromatin remodeling also contributes directly or indirectly to the FoxO-dependent atrophy program. The bromodomain and extra terminal domain (BET) protein BRD4 has been recently shown to be an epigenetic regulator of muscle mass^80^. Mechanistically, BET proteins promote an IL6-AMPK-FoxO3 response to enhance protein breakdown. Foxk is another chromatin remodeling factor that acts as a repressor. When nutrients are available, the mTORC1 complex promotes nuclear relocalization of Foxk, which binds to the promoters of autophagy genes, recruits the Sin3A-HDAC complex, and compacts chromatin, therefore preventing FoxO3 recruitment. In the absence of nutrients, Foxk is in cytosol allowing FoxO3-dependent transcription of autophagy genes^81^.

### Novel and emerging pathways that need consolidation: NF-kB regulation

NF-kB activation has been linked to long non-coding RNAs (lncRNA). Gene expression profiling identified Atrolnc-1 as a lncRNA induced in fasting, cancer cachexia, and chronic kidney disease. Expression of Atrolnc-1 promotes protein breakdown while its inhibition attenuates muscle loss in chronic kidney failure. Mechanistically, Atrolnc-1 modulates an inhibitor of NF-kB named A20-binding inhibitor of NF-kB (ABIN-1) resulting in increased MuRF1 expression when this lncRNA is overexpressed^82^.

### Novel and emerging pathways that need consolidation: metabolic regulation

It has been reported that excessive or defective mitochondrial removal via mitophagy causes muscle loss. For instance, the cytokine FGF21 promotes muscle loss by increasing Bnip3-dependent mitophagy^27^ while the absence of casein kinase 2 (CK2) prevents the import of PINK1 inside the mitochondrial intermembrane space via TOM22 resulting in PINK1 accumulation, which triggers Parkin recruitment and mitophagy. However, autophagy flux is impaired in CK2 knockout leading to the formation of p62-positive protein aggregates and myopathy^83^.

Finally, key enzymes in ATP production such as pyruvate dehydrogenase kinase (PDK)4 have been recently reported to be induced in cancer, starvation, diabetes, and sepsis, and its overexpression promotes myofiber shrinkage while its inhibition blunts atrophy^84^.

## Nonautologous signaling that controls muscle mass

In the last years, a new concept is emerging that considers events surrounding muscle fibers as active players in the atrophy process. Indeed, the NF-kB-dependent inappropriate activation of the self-renewing factor Pax7 in muscle stem cells (satellite cells) contributes to myofiber atrophy^85^. Consistently, the activation of TGFβ signaling in muscle stem cells upregulates the Twist-1 transcription factor, which enhances myostatin expression. The induction of myostatin in satellite cells and its secretion induces a secondary Smad2/3-dependent expression of Twist is adult myofibers that by synergizing with the Smads activates the atrophy program^86^ (Fig. 5). Another interstitial cell population that has been recently shown to contribute to muscle wasting after denervation is Fibro-Adipogenic Progenitors (FAPs). The FAPs in denervated muscles activate an inflammatory response program and become the most abundant source of IL6. Consequently, the IL6-STAT3 axis is activated both in FAPs and myofibers leading to atrophy^87^ (Fig. 5).

Another source of atrophic signals comes from extracellular vesicles (EV). Recently, tumor-derived EVs deliver HSP70 and HSP90 to myofibers, where they activate Toll-like receptor 4 (TLR4) and muscle catabolism^88^ (Fig. 5).

Altogether these findings suggest that several signals inside and outside the myofiber act synergistically to control protein breakdown during muscle atrophy.

## Future directions and therapeutic perspective

Despite the great effort of scientists and pharmaceutical companies to identify effective drug targets and chemical compounds to counteract muscle loss, successful pharmacological treatments for atrophying muscle are absent in the clinic. Several pathways have been targeted and a brief description of the effects is listed below.

### Exercise mimetics

While sedentary behavior has a deleterious effect on human health that has been quantified to be similar to smoking and obesity, longitudinal studies have shown that regular exercise extends life expectancy and reduces morbidity in aging^89,90^. AMPK and PGC1α are critical for the beneficial metabolic adaptations of exercise. Currently, there are no modulators of PGC1α, whereas compounds that activate AMPK, like 5-aminoimidazole-4-carboxamide riboside (AICAR), are available. Despite the fact that AICAR has been recently shown to counteract muscle loss in inflammatory diseases such as cancer cachexia and sepsis^91^, it can also elicit side effects due to the connection with FoxOs, mTOR, and ULK1. In fact, the overactivation of AMPK has been linked to cachexia^80^. Possibly, the beneficial effects of AMPK modulators rely on the type of disease and the duration of the treatment.

SIRT1 is a member of the sirtuin family of class III NAD + -dependent protein deacetylases that is induced by physical activity and modulates the acetylation status and function of several stress-dependent genes, such as PGC1α and FoxOs. SIRT1 is activated by rising levels of NAD + , which typically happens during energy stress conditions like exercise. Recently, treatment with nicotinamide mononucleotide, which enhances NAD + production, has been shown to improve mitochondrial respiration and to prevent aging-associated gene expression changes in muscles of mice and humans^92^.

### Myostatin/TGFβ/activin scavengers or BMP agonist

Myostatin/TGFβ signaling is emerging as a critical pathway for the control of muscle mass and therefore has been a target for the development of different inhibitors. Follistatin, antibodies anti-myostatin or its receptor, recombinant myostatin pro-peptide as well as soluble (decoy) ActIIB receptor have been successfully tested in different animal models of muscle loss. However, myostatin inhibitors display important side effects when tested in humans^93^ or display no significant improvement of survival in cancer patients^94^.

Therefore, alternative and more specific approaches to target TGFβ signaling are needed. The recent data that BMP ligands control muscle mass open the possibility to modulate this branch of the TGFβ pathway to counteract muscle loss.

### IGF1 signaling

In adult mammals, IGF1 is synthesized predominately in the liver under the growth hormone (GH) action and acts as a systemic growth factor. However, IGF1 is also produced in extrahepatic tissues where it plays a predominantly autocrine/paracrine role. Several concerns preclude the use of IGF1 or AKT mimetics because they present with potential oncogenic effects. Therefore, compounds/factors that enhance endogenous muscle IGF1 expression have been searched as an alternative route for AKT activation. Among these, ghrelin is a hormone secreted from the stomach, which, by acting on the hypothalamus and the pituitary gland, induces GH-IGF1 axis and reduces atrophy^95^. Consistently, anamorelin, an oral ghrelin-receptor agonist, elicits an anabolic action in patients with cancer anorexia–cachexia syndrome^96^.

Modulation of FoxOs by altering their acetylation status is another strategy to reduce atrogenes induction. Indeed, HDAC inhibitors have been shown to be successful in blocking FoxO and sparing muscle mass^65^.

### Autophagy and mitophagy modulators

The autophagy system has a dual role: it contributes to muscle loss when hyperactivated, and promotes muscle degeneration when blocked. Specific inhibitors of autophagy are still lacking and the ones (e.g., bafilomycin, chloroquine) that are used in vitro and in vivo mainly block lysosomal function. Conversely, autophagy activators have been developed and are more specific than inhibitors. For instance, the peptide Tat-beclin1 enhances beclin1 function (Fig. 3) by releasing the inhibitory factor GAPR-1 and consequently, increases autophagy flux. Treatment with Tat-Beclin1 in inducible muscle-specific Raptor knockout mice, which showed autophagy impairment and several features of sarcopenia such as mitochondrial dysfunction, atrophy, and denervation, restored a normal mitochondrial membrane potential and reduced myofiber denervation^97^. Recently, urolithin A, a metabolite of ellagitannins that are abundant in pomegranate fruit, as well as in nuts and berries was shown to selectively activate mitophagy in muscle and improve muscle function in aging. Urolithin A was found to activate mitophagy via PINK1/parkin and BNIP3 pathways^56^.

Acetylation of autophagy proteins plays an inhibitory action, and the polyamine spermidine, by blocking the EP300 acetyltransferase, prevents this post-translational modification, maintains autophagy flux, preserves mitochondrial function, exhibits anti-inflammatory properties, and prevents stem cell senescence^98^.

### β2-adrenoreceptor agonists

Clenbuterol or formoterol are β2-adrenoreceptor agonists that in addition to stimulating the breakdown of glycogen and lipids, enhance protein synthesis and inhibit protein degradation^25^. There is some reluctancy in the muscle field to use β2-adrenoreceptor agonists for their possible side effects on heart function, especially for cardiac arrhythmias. However, recent clinical trials on Pompe patients (lysosomal storage diseases disorder) with clenbuterol did not report any serious adverse events^99^. Alternative, inhibitors of phosphodiesterase 4 (PDE4) increase cAMP and activate PKA like β2-adrenoreceptor agonists.

### Inhibitors of ubiquitin–proteasome system

Several genetic evidences underline the critical role of ubiquitin–proteasome in degrading sarcomeric proteins. Interestingly, a specific inhibitor of the muscle-specific ubiquitin ligase MuRF1 was developed and a protective effect in atrophying muscles of mice with heart failure was found^100^.

## Concluding remarks

Substantial progress has been made in our understanding of the molecular mechanisms that mediate the loss of muscle mass in disease. The lack of any efficient drug that counteracts muscle loss suggests that our view of the mechanistic insights that control atrophying muscle is still limited and needs further exploration.
